# Effects of Bariatric Surgery on Renal Function in Obese Patients: A Systematic Review and Meta Analysis

**DOI:** 10.1371/journal.pone.0163907

**Published:** 2016-10-04

**Authors:** Kun Li, Jianan Zou, Zhibin Ye, Jianzhong Di, Xiaodong Han, Hongwei Zhang, Weijie Liu, Qinggui Ren, Pin Zhang

**Affiliations:** 1 Department of General Surgery, Huadong Hospital Affiliated to Fudan University, Shanghai, China; 2 Department of Nephrology, Huadong Hospital Affiliated to Fudan University, Shanghai, China; 3 Department of General Surgery, Shanghai Jiao Tong University Affiliated Sixth People’s Hospital, Shanghai, China; University Medical Center Utrecht, NETHERLANDS

## Abstract

**Background:**

Obesity is an independent risk factor of development and progression of chronic kidney disease (CKD). Data on the benefits of bariatric surgery in obese patients with impaired kidney function have been conflicting.

**Objective:**

To explore whether there is improvement in glomerular filtration rate (GFR), proteinuria or albuminuria after bariatric surgery.

**Methods:**

We comprehensively searched the databases of MEDLINE, Embase, web of science and Cochrane for randomized, controlled trials and observational studies that examined bariatric surgery in obese subjects with impaired kidney function. Outcomes included the pre- and post-bariatric surgery GFR, proteinuria and albuminuria. In obese patients with hyperfiltration, we draw conclusions from studies using measured GFR (inulin or iothalamate clearance) unadjusted for BSA only. Study quality was evaluated using the Newcastle-Ottawa Scale.

**Results:**

32 observational studies met our inclusion criteria, and 30 studies were included in the meta-analysis. No matter in dichotomous data or in dichotomous data, there were statistically significant reduction in hyperfiltration, albuminuria and proteinuria after bariatric surgery.

**Limitations:**

The main limitation of this meta-analysis is the lack of randomized controlled trials (RCTs). Another limitation is the lack of long-term follow-up.

**Conclusions:**

Bariatric surgery could prevent further decline in renal function by reducing proteinuria, albuminuria and improving glomerular hyperfiltration in obese patients with impaired renal function. However, whether bariatric surgery reverses CKD or delays ESRD progression is still in question, large, randomized prospective studies with a longer follow-up are needed.

## Introduction

Obesity is a growing problem in the world and is associated with highly elevated risks of adverse health outcomes. The Non-Communicable Diseases Risk Factor Collaboration revealed that between 1975 and 2014 the prevalence of obesity increased from 3.2% to 10.8% in men and from 6.4% to 14.9% in women in their pooled analysis of 1698 population-based studies including more than 19 million participants [1]. Also, obesity is a strong trigger of diabetes mellitus (DM), dyslipidemia, hypertension and metabolic syndrome which are strong risk factors for the development and progression of chronic kidney disease (CKD)[2, 3].

Bariatric surgery has been approved as an effective treatment that achieves dramatic and durable weight loss in obese patients [4]. Several studies have shown impressive improvements in hypertension, dyslipidemia as well as diabetic complications following bariatric surgery [5, 6]. However, the effects of weight loss and improved metabolic disorder on renal diseases after bariatric surgery have been poorly evaluated.

Extreme obesity is responsible for glomerulosclerosis [7]. Renal diseases in the setting of obesity often manifest albuminuria, proteinuria, glomerular hyperfiltration and decreased glomerular filtration rate (GFR)[8, 9]. Although many retrospective studies have shown improvement in proteinuria and impaired GFR, results vary in effect size, type of outcome, and precision. Several systematic reviews explored the effects of dietary restriction, weight-loss drug or exercise on renal function in obese subjects with or without CKD [10–12], and some meta-analysis researched the effects of bariatric surgery on albuminuria, proteinuria included cohorts with either normal range, nephrotic range or both [13, 14]. Also, some reviews only reported descriptive outcomes from each study without calculating a pooled effect size of proteinuria and impaired GFR. Thus, to quantitatively summarize existing evidences regarding the effects of bariatric surgery on nephrotic range albuminuria, proteinuria and impaired GFR, we performed a systematic review and meta-analysis of observational studies to find whether bariatric surgery could ameliorate nephrotic range albuminuria or proteinuria and reverse hyperfiltration or hypofiltration in obese individuals with impaired renal function.

## Materials and Methods

### Study Design

A systematic review and meta-analysis was conducted according to predefined guidelines provided by the Cochrane Collaboration (2008)[15]. All data were reported according to Meta-analysis Of Observational Studies in Epidemiology statement [16].

### Search Strategy

Two authors (Kun Li, Jianan Zou) independently searched published studies indexed in the MEDLINE, EMBASE, web of science and the Cochrane Central Register of Controlled Trials (CENTRAL) in The Cochrane Library. References of all selected studies were also examined. The following main search terms were used: bariatric surgery, gastric bypass, sleeve gastrectomy, gastroplasty, biliopancreatic diversion, weight loss, kidney disease, obese, albuminuria, proteinuria, microalbuminuria, macroalbuminuria, renal function, glomerular filtration rate and creatinine. The latest date for this search was March, 2016.

### Inclusion and exclusion criteria

This review included all published randomized controlled trials or observational studies including cohort, cross-sectional, and case-control studies that assessed the effects of bariatric surgery on impaired renal function in obese patients. Reviews, case reports, abstracts, and unpublished studies were excluded.

Two reviewers (Kun Li, Jianan Zou) independently screened all abstracts and selected studies in the meta-analysis if they met all of the following criteria: (1) randomized, controlled trial (RCT) or observational study; (2) minimum intervention period of 4 weeks; (3) studies aimed to analyze the impact of bariatric surgery in obese patients with hypofiltration; (4) studies that analyzed the effects of bariatric surgery in obese patients with micro- or macroalbuminuria or proteinuria and (5) studies that analyzed the impact of bariatric surgery on GFR in obese patients with glomerular hyperfiltration; (6) reports of pre- and post-surgery mean values (if not available, change from baseline values were used) with standard deviation (or basic data to calculate these parameters: standard error, 95% confidence interval, p-values). If data of ongoing studies were published as updates, results of only the longest duration periods were included. For studies without the outcomes we needed, author(s) would be contacted via e-mail for more relevant information, if necessary. In studies that analyzed multiple interventions, only data conducted by bariatric surgery were considered for inclusion.

All studies analyzing glomerular hyperfiltration were divided into four subgroups: mGFR, CrCl, eGFR with and without BSA, and they were analysed separately. Serum creatinine varies with both GFR and muscle mass, so eGFR and CrCl are influenced by both true GFR and muscle mass. The use of serum creatinine-based equations is problematic following bariatric surgery. In addition, eGFR and mGFR values adjusted for BSA lead to a systematic underestimation of GFR in patients with severe obesity [17]. Thus CrCl, eGFR with and without BSA are all clearly unreliable. In our review, we draw conclusions from studies using measured GFR (inulin or iothalamate clearance) without adjusted for BSA only.

Renal function impairment was considered as the stable presence of one or more of the following conditions: (i) GFR <90 mL/min (hypofiltration) (ii) GFR >125 mL/min (hyperfiltration), (iii) pathological proteinuria or albuminuria. As long as the population in the studies fulfilled the above criteria, they were included in this review. Obesity was defined as BMI >30 kg/m^2^ and hyperfiltration was defined as GFR>125 mL/min. GFR between 60 and 90 mL/min was considered as slightly impaired glomerular function [18]. Albuminuria was classified as microalbuminuria and macroalbuminuria. Microalbuminuria is defined as urinary albumin-to-creatinine ratio (UACR) between 30 and 300 mg/g of creatinine or 24-h albuminuria between 30 and 300 mg. Macroalbuminuria is defined as UACR>300 mg/g of creatinine or 24-h albuminuria>300 mg/g. 24-h proteinuria>0.15 g and 24-h albuminuria>30 mg were considered pathologic range.

Exclusion criteria were (1) reviews, comments, case reports and case series, (2) studies that analyzed the effects of bariatric surgery in dialysis patients, and (3) studies that assessed the impacts of bariatric surgery on albumin excretion in obese subjects with normal albuminuria. In studies that enrolled both patients with normal GFR and impaired GFR, only data relating to impaired GFR were included in the analysis. Similarly, in studies that enrolled both patients with normal albuminuria and microalbuminuria, only data pertaining to patients with microalbuminuria (when available) were extracted.

### Data extraction

Two investigators (Kun Li, Jianan Zou) independently reviewed abstracts of all citations. Data verifications between the two authors were performed to ensure reliability and completeness after all abstracts were reviewed. The inclusion criteria were applied to all identified studies independently. Different decisions were resolved by consensus.

Full texts of potentially relevant articles identified through other sources were retrieved. If multiple articles from the same study were searched, only the article with the longest follow-up period was included. Data with respect to research design, type of surgery, participant characteristics, duration of study, and outcome were independently extracted. We contacted the authors for the primary reports of the unpublished data. If the authors did not reply, the available data were used for our analyses.

### Methodological Quality Assessment

We used the nine-point Newcastle-Ottawa Scale to assess the study quality for all included observational studies. This scale evaluated a quality score calculated on three fundamental methodological criteria: study participants (0–4), adjustment for confounding (0–2) or ascertainment of the exposure or outcome of interest (0–3). We arbitrarily classified quality as high (score: 7–9) versus low (score: 0–3). We excluded studies from our meta-analysis if they had poor quality. Discrepant opinions between authors were resolved to reach a consensus.

### Statistical Analysis

The data were pooled using REVMAN 5.0 software (The Nordic Cochrane Centre, Copenhagen, Denmark). For each study, we calculated Relative Risk (RR) with 95% Confidence Intervals (CIs) for dichotomous data and Standardised Mean Difference (SMD) with 95% CIs for continuous data. A Random-effect model (DerSimonian-Laird method) was used when significant heterogeneity was detected between studies (*P*<0.10; I^2^>50%). Otherwise, a Fixed-effect model (Mantel-Haenszel test) was used. To assess the stability of the results of the meta-analysis, sensitivity analysis was performed. Publication bias was assessed by the Egger’s test and represented graphically by funnel plots.

## Results

### Description of included studies

**Fig 1:**
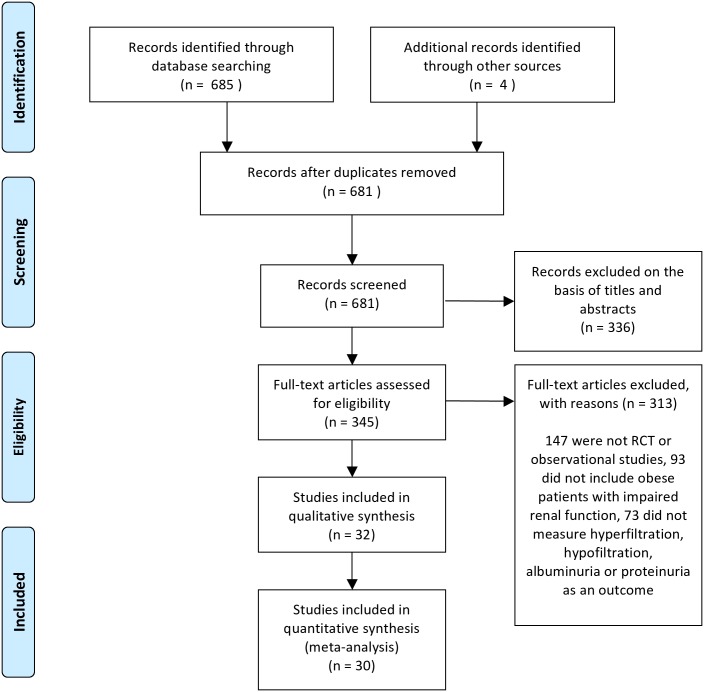
Flow diagram of the selection process. RCT: randomized, controlled trial.

After excluding duplicate results, the initial search included 681 articles, 661 articles were excluded because 336 were off the topic after scanning the title and/or the abstract, 147 were not RCT or observational studies, 93 did not include obese patients with impaired renal function, and 73 did not measure hyperfiltration, hypofiltration, albuminuria or proteinuria as an outcome. 32 observational studies met our inclusion criteria, and 30 studies were included in the meta-analysis (Fig 1) and the characteristics are outlined in Table 1.

**Table 1:** Study details and patient demographics.

Study	Type of study	No. of patients (female)	Age (years)	Baseline BMI (kg/m^2^)	Baseline kidney disease included in our study	Baseline kidney disease excluded in our study	Inventions	Follow-up (months)	Renal outcomes	GFR adjusted or unadjusted for BSA
**Brøchner 1980[19]**	Prospective cohort	8(7)	26–40	136.4	Glomerular hyperfiltration	-	Intestinal bypass surgery	12	mGFR(EDTA)	unadj/BSA
**Chagnac 2003[20]**	Prospective cohort	8(4)	36 ± 2	48.0±2.4	Glomerular hyperfiltration	-	Gastroplasty	12	mGFR(inulin clearance)	unadj/BSA
**Agrawal 2008[21]**	Retrospective	94(72)	45.5 ± 10	49.1 ± 8	Microalbuminuria	Macroalbuminuria	RYGB	12	ACR; SCrSCr;	-
**Navaneethan 2009[22]**	Retrospective	25(18)	51.5± 7.4	49.8±7.5	CKD III	Acute renal failure	Bariatric surgery	12	eGFR(MDRD); SCrSCr;	adj/BSA
**Serpa 2009[23]**	Retrospective	140(96)	18–60	46.1 ± 5.4	Glomerular hyperfiltration; Proteinuria; AlbuminuriaProteinuria; Albuminuria	-	RYGB	8	CrCl; proteinuria; albuminuriaProteinuria; albuminuria	unadj/BSA
**Amor 2013[24]**	Observational prospective study	255	45.6± 10.6	47.7 ± 6	Microalbuminuria	Patients with proteinuria in the nephrotic range, previously diagnosed with glomerulonephritis, with eGFR<60 ml/min, or with history of renal transplantation at baseline	RYGB; SGSG	24	ACR; SCrScr	-
**Fenske 2013[25]**	Prospective observational study	34(29)	35–54	44.6±.9	CKD II; AlbuminuriaAlbuminuria	eGFR<60 mL/min/1.73 m2	AGB; RYGB; SGRYGB; SG	12	eGFR(MDRD); SCr; ACRScr; ACR	adj/BSA
**Hou 2013[26]**	Retrospective	233(184)	33.1 ±9.7	39.5± 9.7	Glomerular hyperfiltration; CKD II; CKD III; Microalbuminuria; MacroalbuminuriaCKD III; Microalbuminuria; Macroalbuminuria	-	AGB; SG; RYGB; mini-gastric bypassSG; RYGB; mini-gastric bypass	12	eGFR(MDRD); ACR; UCrACR; Ucr;	unadj/BSA
**Stephenson 2013[27]**	Retrospective	23(11)	58 ±9	40.1 ± 5.4	Microalbuminuria; Macroalbuminuria	-	LAGB	36	ACR;	-
**Ruiz-Tovar 2014[28]**	Prospective observational	50(44)	49.2±6.4	48.4±7.7	CKD II	eGFR<60 ml/min/1.73^2^	LSG	12	eGFR(MDRD); SCr; UCrScr; Ucr	adj/BSA
**Kim 2015[29]**	Prospective consecutive	136(101)	35.9_ ±11.2	39.9± 6.3	Microalbuminuria; Glomerular hyperfiltrationGlomerular hyperfiltration	significant chronic kidney disease, macroalbuminuria, nephrotic range proteinuria	RYGB; SGSG	12	eGFR; ACR; PCRACR; PCR	adj/BSA
**Miras 2015[30]**	Prospective casde—control	70(53)	50.7± 1.0	43.6 (40.6–49.7)	Albuminuria	-	RYGB	12	ACR	-
**Ngoh 2015[31]**		68	40.7±10.8	41.9± 5.7	Glomerular hyperfiltration; CKD II; CKD IIICKD II; CKD III	-	Gastric bypass; SGSG	12	eGFR; CrclCrcl	unadj/BSA
**Navarro-Diaz 2006[32]**	Prospective	61(37)	41.10 ±9.07	53.62± 9.65	Microalbuminuria; Proteinuria; Glomerular hyperfiltrationProteinuria; Glomerular hyperfiltration	-	Gastric bypass	24	Crcl; Scr; 24h-proteinuria; 24h-albuminuriaScr; 24h-proteinuria; 24h-albuminuria	unadj/BSA
**Reid 2014[33]**	Retrospective	158(145)	48.8± 0.9	47.0± .6	Microalbuminuria; Glomerular hyperfiltrationGlomerular hyperfiltration	CKD>Stage 3; Macroalbuminuria	RYGB; SGSG	12	Crcl; Scr; ACR; eGFR(CG-LBW)Scr; ACR; eGFR(CG-LBW)	unadj/BSA
**Palomar 2005[34]**	Prospective	35(29)	40.1 ± 11.6	46.9 ± 6.3	Albuminuria	-	BPD	12	ACR; 24h-albluminuria24h-albluminuria	-
**Lieske 2014[35]**	Prospective cohort study	11(11)	49.5±11.5	45.7±5.0	Glomerular hyperfiltration	-	RYGB; BPDBPD	12	mGFR (iothalamate clearance); eGFR(CKD-EPI); Crcl; ScreGFR(CKD-EPI); Crcl; Scr	unadj/BSA
**Zhang 2015[36]**	Retrospective	101	DN3: 47.6±13.7, DN4: 44.1±8.7	DN3: 31.7±3.9, DN4: 31.7±3.2	T2DM with DN3 and DN4	-	RYGB	12	mGFR(99mTc-DTPA); ACR; 24h-albluminuria24h-albluminuria	unadj/BSA
**Mohan 2012[37]**	Cohort	38(34)	41 ± 10.3	46±8	Microalbuminuria	-	RYGB	30 days	ACR	-
**Celik 2013[38]**	Retrospective	33(31)	45.2±8.5	44.6±5.4	Microalbuminuria	-	RYGB	21	ACR; 24h-albluminuria24h-albluminuria	-
**Gonzalez-Heredia 2016[39]**	Non-randomized, Controlled Retrospective	30(28)	52.6± 10.9	51.6± 9.3	CKD II; CKD IIICKD III	-	SG	6	Crcl(CG)	adj/BSA
**Zakaria 2015[40]**	Retrospective	20	44.7± 9.5	42.8±4.9	CKD II; CKD IIICKD III	-	AGB	13.8± 2.04 years	eGFR	adj/BSA
**Friedman 2014[41]**	Retrospective	36(28)	50±11	46±9	Glomerular hyperfiltration	serum creatinine level>.1.3 mg/dL for women and>.1.5 mg/dL for men, and dialysis dependency	Bariatric surgery	296±103 days	mGFR	unadj/BSA
**Heneghan 2013[42]**	Retrospective cohort	52(39)	51.2± 10.1	49±8.7	Albuminuria	-	RYGB; SG; AGBSG; AGB	66	24h-albluminuria	-
**Navaneethan 2010[43]**	Pilot study	15(6)	51± 14	49±9	Microalbuminuria	-	RYGB;other types of bariatric surgery other types of bariatric surgery	6	ACR; ScrScr	-
**Abouchacra 2013[44]**	Retrospective cohort	220(145)	34.7 ± 10	47±9	Glomerular hyperfiltration; CKD IICKD II	eGFR<60 ml/min; chronic nephrotoxic medication use; underlying chronic illness or malignancy	Bariatric surgery	6	Crcl(CG-LBW); eGFR(MDRD,CKD-EPI)eGFR(MDRD,CKD-EPI)	Both adj/BSA and unadj/BSA
**Iaconelli 2011[45]**	Case-control	22(12)	43.8 ± 8.3	142.5±29.3	Microalbuminuria		BPD	120	24h-albuminuria	-
**Kumar 2009[46]**	Prospective	10(6)	48.2± 9	33.8± 6.5	Microalbuminuria	eGFR<60 ml/min	SG; ileal interpositionileal interposition	9.1±5.3	24h-albuminuria	-
**Kota 2011[47]**	Prospective cohort	38(14)	47.5 ± 8.8	32.05 ± 7.5	Albuminuria	-	Laparoscopic leal inter Position(II) with sleeve gastrectomy (SG)	11.3 ± 9	24h-albuminuria	-
**Miras 2012[48]**	Retrospective cohort	84	50.2±1.1	47.5±0.8	Patients with T2DM; Albuminuria 42.7%Albuminuria 42.7%	-	LRYGB, gastric banding, SG	12–18	ACR	-
**Zeve 2013[49]**	Prospective cohort	17(10)	Mean 44.9	44.3 ± 1.3	Microalbuminuria	-	LRYGB	12	Crcl; Proteinuria; MicroalbuminuriaProteinuria; Microalbuminuria	unadj/BSA
**Saliba 2010[50]**	cohort	35(32)	45 ± 9	47 ± 8	Proteinuria; MicroalbuminuriaMicroalbuminuria	-	RYGB	12	Crcl; Scr; Proteinuria; MicroalbuminuriaScr; Proteinuria; Microalbuminuria	unadj/BSA

CKD 2: chronic kidney disease stages II; CKD 3: chronic kidney disease stages III; DN3: Diabetic Nephropathy stages III; DN4: Diabetic Nephropathy stages IV; T2DM: type 2 diabetes mellitus, AGB: Adjustable Gastric Band; SG: Sleeve Gastrectomy; BPD: Biliopancreatic Diversion; RYGB: Roux-en-Y Gastric Bypass; LAGB: Laparoscopic Adjustable Gastric Band; LSG: Laparoscopic Sleeve Gastrectomy; LRYGB: Laparoscopic Roux-en-Y Gastric Bypass; mGFR: measured glomerular filtration rate; eGFR: estimated glomerular filtration rate; Crcl: creatinine clearance; Scr: Serum creatinine;ACR: albumin-to-creatinine ratio; PCR: protein-to-creatinine ratio; CKD-EPI: Chronic Kidney Disease Epidemiology Collaboration equation; CG-LBW: lean weight—adjusted Cockcroft-Gault creatinine clearance; MDRD: Modification of Diet in Renal Disease equation; unadj/BSA: unadjusted for BSA; adj/BSA: adjusted for BSA.

### Quality assessment of included studies

NOS evaluated the quality of the included studies. Total score ranged from 4 to 8. None of the studies had low quality (total score below 3) and excluded from the meta-analysis.

### Meta-analysis results

Due to their outcomes could not be combined with other studies, 2 studies [41, 48] were excluded from meta-analysis. 14 studies of 1186 patients with dichotomous data [21, 23, 24, 26, 27, 29, 32–34, 38, 42, 43, 45, 51] and 10 studies of 930 patients with continuous data [24, 25, 29, 33, 36, 37, 46, 47, 49, 50] were included in the meta-analysis of albuminuria and proteinuria. There were only 5 studies of 184 patients [25, 26, 28, 40, 44] in the review of CKD II. Due to 3 studies [26, 39, 43] using CrCl, eGFR with and without adjustment for BSA, the continuous data could not be combined in the meta-analysis of CKD III. Furthermore, 9 studies of 631 patients with continuous data [19, 20, 23, 26, 31, 32, 35, 44, 52] and 6 studies of 514 patients with dichotomous data [23, 29, 32, 33, 35, 53] were included in the meta-analysis of hyperfiltration.

**Fig 2:**
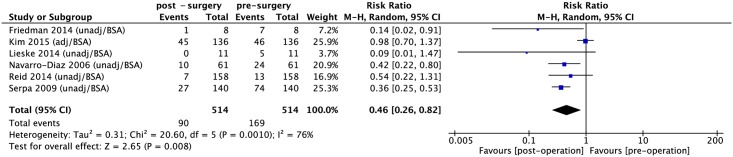
Forest plot comparing glomerular hyperfiltration (dichotomous data) between presurgery and postsurgery. unadj/BSA: unadjusted for BSA; adj/BSA: adjusted for BSA.

**Fig 3:**
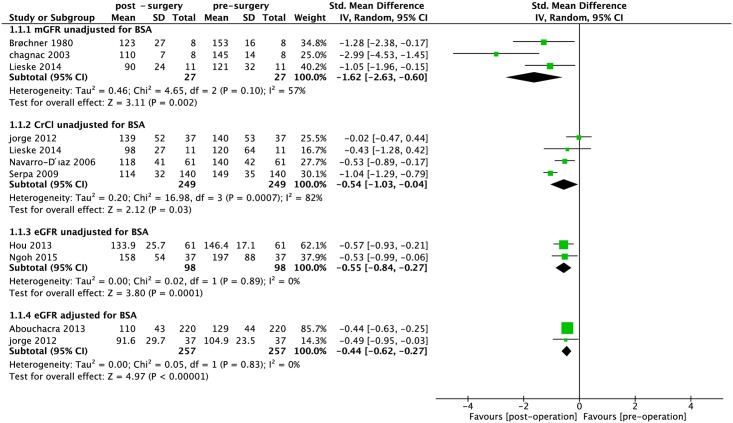
Forest plot comparing glomerular hyperfiltration (continuous data) between presurgery and postsurgery. mGFR: measured glomerular filtration rate; eGFR: estimated glomerular filtration rate; Crcl: creatinine clearance; BSA: body surface area; unadj/BSA: unadjusted for BSA; adj/BSA: adjusted for BSA.

The dichotomous data presented in Fig 2 show there was a statistically significant reduction in hyperfiltration after bariatric surgery (RR: 0.46, 95% CI 0.26–0.82, *P* = 0.008; I^2^ = 76%; *P*_heterogeneity_ = 0.001) (Fig 2). The continuous data presented in Fig 3 were divided into four subgroups. Meta-analysis showed statistically significant decrease in mGFR, CrCl, eGFR with and without adjustment for BSA after bariatric surgery (SMD: -1.62, 95% CI: -2.63 –-0.60, *P* = 0.002; I^2^ = 57%; *P*_heterogeneity_ = 0.1; SMD: -0.54, 95% CI: -1.03 –-0.04, *P* = 0.03; I^2^ = 82%; *P*_heterogeneity_ = 0.0007; SMD: -0.55, 95% CI: -0.84 –-0.27, *P* = 0.0001; I^2^ = 0%; *P*_heterogeneity_ = 0.89; SMD: -0.44, 95% CI: -0.62 –-0.27, *P*< 0.0001; I^2^ = 0%; *P*_heterogeneity_ = 0.83; respectively) (Fig 3).

**Fig 4:**
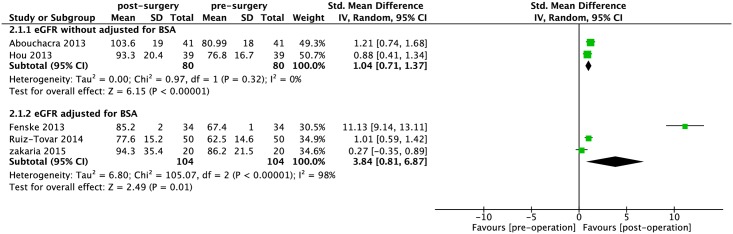
Forest plot comparing CKD II (continuous data) between presurgery and postsurgery. eGFR: estimated glomerular filtration rate; BSA: body surface area.

Likewise, we found statistically significant increase in eGFR with and without adjustment for BSA after bariatric surgery (SMD: 1.04, 95% CI: 0.71–1.37, *P*< 0.0001; I^2^ = 0%; *P*_heterogeneity_ = 0.32; SMD: 3.84, 95% CI: 0.81–6.87, *P* = 0.01; I^2^ = 98%; *P*_heterogeneity_< 0.0001) (Fig 4).

**Fig 5:**
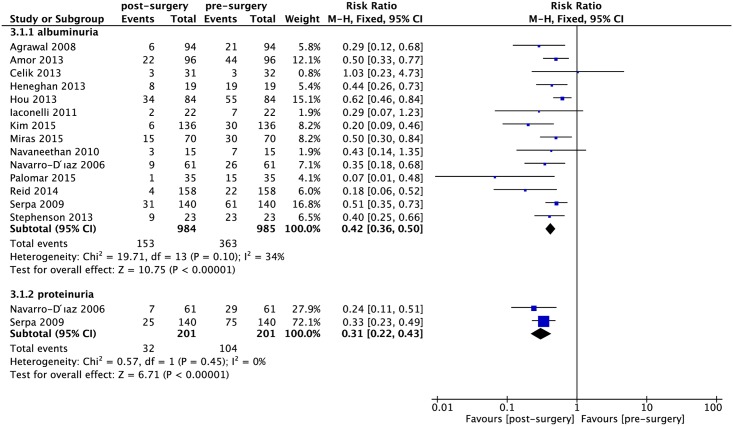
Forest plot comparing albuminuria and proteinuria (dichotomous data) between presurgery and postsurgery.

**Fig 6:**
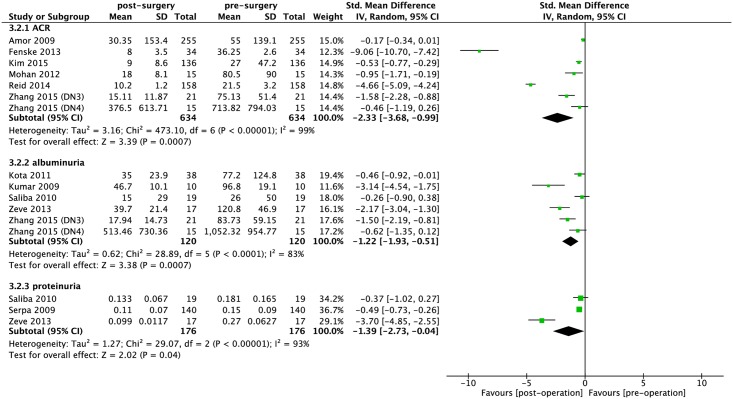
Forest plot comparing albuminuria and proteinuria (continuous data) between presurgery and postsurgery. DN3: Diabetic Nephropathy stages III; DN4: Diabetic Nephropathy stages IV; ACR: albumin-to-creatinine ratio.

There was a statistically significant reduction in the incidence of albuminuria and proteinuria after bariatric surgery (RR: 0.42, 95% CI: 0.36–0.50, *P*< 0.0001; I^2^ = 34%; *P*_heterogeneity_ = 0.10; RR: 0.31, 95% CI: 0.22–0.43, *P*< 0.0001; I^2^ = 0%; *P*_heterogeneity_ = 0.45; respectively) (Fig 5). In addition, the continuous data were presented in Fig 6. Meta-analysis showed statistically significant decrease in ACR and 24-h albuminuria after bariatric surgery (SMD: -2.33, 95% CI: -3.68 –-0.99, *P* = 0.0007; I^2^ = 99%; *P*_heterogeneity_< 0.0001; SMD: -1.22, 95% CI: -1.93 –-0.51, *P* = 0.0007; I^2^ = 83%; *P*_heterogeneity_< 0.0001; respectively) (Fig 5). Furthermore, there is statistically significant decrease in proteinuria after bariatric surgery (SMD: -1.39, 95% CI: -2.73 –-0.04, *P* = 0.04; I^2^ = 93%; *P*_heterogeneity_< 0.0001) (Fig 6).

### Sensitivity analysis

**Fig 7:**
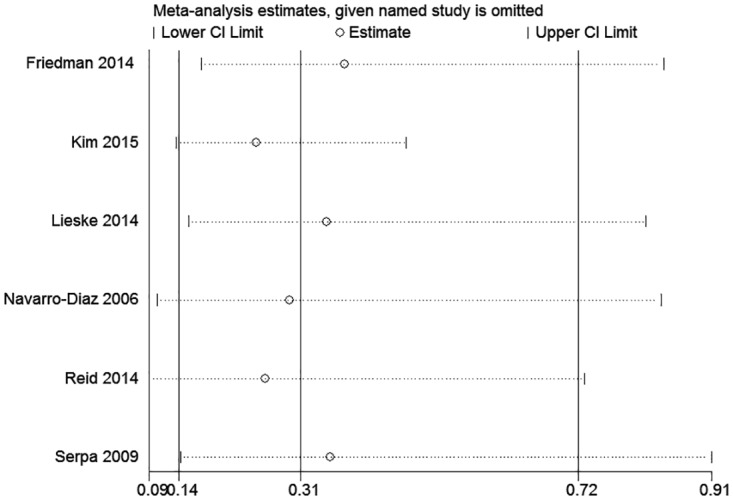
The sensitivity analysis of the results of the meta-analysis of the effect of bariatric surgery on glomerular hyperfiltration.

**Fig 8:**
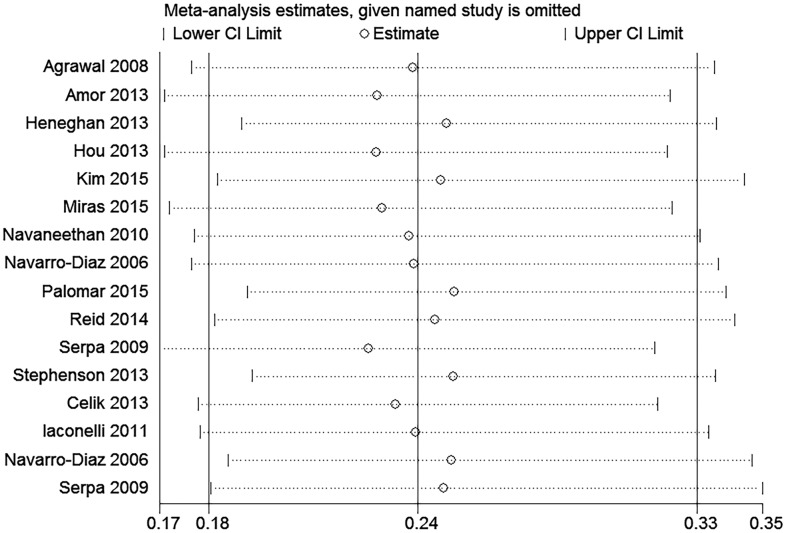
The sensitivity analysis of the results of the meta-analysis of the effect of bariatric surgery on albuminuria and proteinuria.

To assess the stability of the results of the meta-analysis of hyperfiltration, albuminuria and proteinuria, sensitivity analyses were conducted by excluding 1 study at a time. None of the results was significantly altered, indicating that our results were robust (Figs 7 and 8).

### Publication bias

Because publication bias could affect the results of meta-analyses, we attempted to evaluate this potential publication bias by using funnel plots analysis and Egger’s test. Visualizing funnel plots for studies evaluating hyperfiltration, proteinuria and albuminuria, suggested a symmetric distribution of studies around the effect size and the Egger’s test confirmed the lack of publication bias in proteinuria and albuminuria (*P* = 0.562).

## Discussion

The earliest study about the effect of bariatric surgery on renal function was published in 1980. Over the last 3 decades, the outcomes of bariatric surgery in obese patients with regard to mediating sustained weight reduction have been extensively evaluated. It is necessary to conduct a systematic review and meta-analysis assessing the effects of bariatric surgery on improvement of renal parameters in obese patients with impaired renal function. All studies included in our article investigated either the change of glomerular filtration capacity or the reduction in amount of urinary albumin or protein excretion in obese patients after bariatric surgery. Although several studies had relatively small sample size or loss of follow-up, there was statistically significant improvement of all parameters in obese patients with impaired renal function after bariatric surgery. There is a lack of long-term studies that analyzed the impact of bariatric surgery on the progressive of ESRD and mortality.

Obesity has been regarded as an independent risk factor for chronic kidney disease [54–56]. Several studies showed that glomerular hyperfiltration caused by obesity reflected loss of renal functional reserve and contributed to the development and progressive of CKD [57, 58]. Firstly, glomerulomegaly and focal glomerulosclerosis have been closely associated with obesity in order to meet increased metabolic demands in morbidly obese patients. These disorders are characterized by hyperfiltration, which leads to segmental scarring and worsen renal function. Secondly, abnormalities in vascular control associated with afferent renal vasodilation and increased renal blood flow might lead to the development of glomerular hyperfiltration in obese patients with diabetes. In the subgroup analysis of obese patients with glomerular hyperfiltration, using CrCl, eGFR with or without adjusted for BSA, a significant decrease in CrCl and eGFR (adjusted and unadjusted for BSA) was seen after bariatric surgery. However, firm conclusions cannot be drawn due to likely confounding effects of changes in muscle mass and protein intake on serum creatinine. In addition, eGFR and mGFR values adjusted for BSA lead to a systematic underestimation of GFR in patients with severe obesity [17], thus they are clearly unreliable with adjusted for BSA. In our review, we draw conclusions from studies using measured GFR (inulin or iothalamate clearance) unadjusted for BSA only. We found a statistically significant decrease in mGFR, indicating that glomerular hyperfiltration was significantly improved in obese patients after bariatric surgery. However, whether this normalization in hyperfiltration could translate into long-term renal benefits remains to be seen.

The association between obesity and CKD may be mediated through multiple biologic mechanisms. Excess adipose tissue can lead to the activation of the sympathetic nervous and renin-angiotensin systems, as well as lipid deposition, hyperfiltration, and increased sodium absorption in the kidneys, resulting in a feedback loop where obesity-induced declines in kidney function lead to the development of hypertension, which results in further damage to the kidneys [59, 60]. Pathways leading from obesity to diabetes have also been identified, including the development of insulin resistance through the disruption of insulin signaling pathways due to lipolysis, the release of adipokines [61] and inflammation [62]. In the morbidly obese population, weight loss that is attained through bariatric surgery results in an improvement in insulin resistance, oxidative stress, and inflammation [63, 64]. These improvements may contribute to the observed better outcomes after bariatric surgery in obese patients with CKD [25, 28]. As for CKD patients, the perioperative period is a time of considerable increase stress originating from fluid and hemodynamic shifts that can lead to acute kidney failure, and cardiac risk factors including angina, myocardial infarction, congestive heart failure, and DM have an intermediate probability of increased perioperative risk [65]. This may be the main reason why few patients have been included with advanced CKD in observational studies so far published. Several studies suggested that obese patients with CKD II and III could benefit from the improvement of GFR after bariatric surgery [25, 26, 28, 40, 44] and we found statistically significant increase in eGFR postoperatively. Because they used eGFR with or without adjusted for BSA to estimate glomerular filtration capacity, the results were still worth discussing. Inulin clearances have been regarded as the gold standard of GFR. So to assess whether there is a beneficial effect of bariatric surgery on kidney function of CKD patients requires further studies with larger sample size and longer duration of follow-up and GFR must be measured with exogenous glomerular filtration tracers.

Although GFR is the backbone of the current CKD classification and a low GFR is an important risk factor for end-stage renal disease (ESRD) [18], the impact of albuminuria for cardiovascular disease and CKD is significantly remarkable [66]. It is suggested that microalbuminuria was a sign of vascular damage and macroalbuminuria is evidence of a diseased glomerulus, so albuminuria has been considered as an independent risk factor of cardiovascular events and ESRD [67]. Several studies have consistently shown that GFR and ACR complement each other very well and both a higher albuminuria and a lower GFR provide synergistic, complementary risk-stratification for both CKD and cardiovascular disease [66, 68, 69]. Albuminuria comes from diabetes mellitus (DM), thus, remission of diabetes may affect the improvement of renal function after bariatric surgery. Our review revealed that the bariatric surgery could remarkably reduce urinary albumin and protein excretion in obese patients.

The heterogeneity between studies analyzing glomerular hyperfiltration, proteinuria and albuminuria were statistically significant. This heterogeneity was further explored in the sensitivity analysis, which suggested our results were robust. We believed that the observed heterogeneity in our meta-analysis was mainly attributed to differences in population, duration of obesity, study design, follow-up, sample size or co-morbidities.

Our review has some strengths and limitations. Strengths included the comprehensive search method, data extraction and study quality assessment made by two independent reviewers. There are also some limitations in our study. First, although comprehensive search strategies focused on bariatric surgery and a specific population (obese patients with impaired renal function) was implemented, this review is subject to publication bias inevitably. Second, most of the included studies are observational reports, which are of suboptimal quality and subject to selection bias. Third, randomized controlled studies of bariatric surgery compared with non-surgical weight loss or medical intervention are needed. Finally, the effect of bariatric surgery on kidney function of CKD patients requires further studies and GFR must be measured with inulin clearance. Further prospective studies are also needed to measure long-term effects of bariatric surgery in obese patients with impaired renal function.

## Conclusions

In conclusion, bariatric surgery could prevent further decline in renal function by reducing proteinuria, albuminuria and improving glomerular hyperfiltration in obese patients with impaired renal function. However, whether bariatric surgery reverses CKD or delays ESRD progression is still in question, large, randomized prospective studies with a longer follow-up are needed.

## Supporting Information

S1 Fig Funnel plot to assess publication. Funnel plot to assess publication for the most frequently reported outcome glomerular hyperfiltration. mGFR: measured glomerular filtration rate; eGFR: estimated glomerular filtration rate; Crcl: creatinine clearance; BSA: body surface area.
(TIF)

S2 Fig Funnel plot to assess publication. Funnel plot to assess publication for the most frequently reported outcome albuminuria and proteinuria.
(TIF)

S1 PRISMA Checklist PRISMA Checklist. (DOC)
