# Supplementary figures and images for: Effects of Bariatric Surgery on Renal Function in Obese Patients: A Systematic Review and Meta Analysis
Source: PLoS One. 2016 Oct 4;11(10):e0163907. doi: 10.1371/journal.pone.0163907 (PMC5049777; doi:10.1371/journal.pone.0163907)

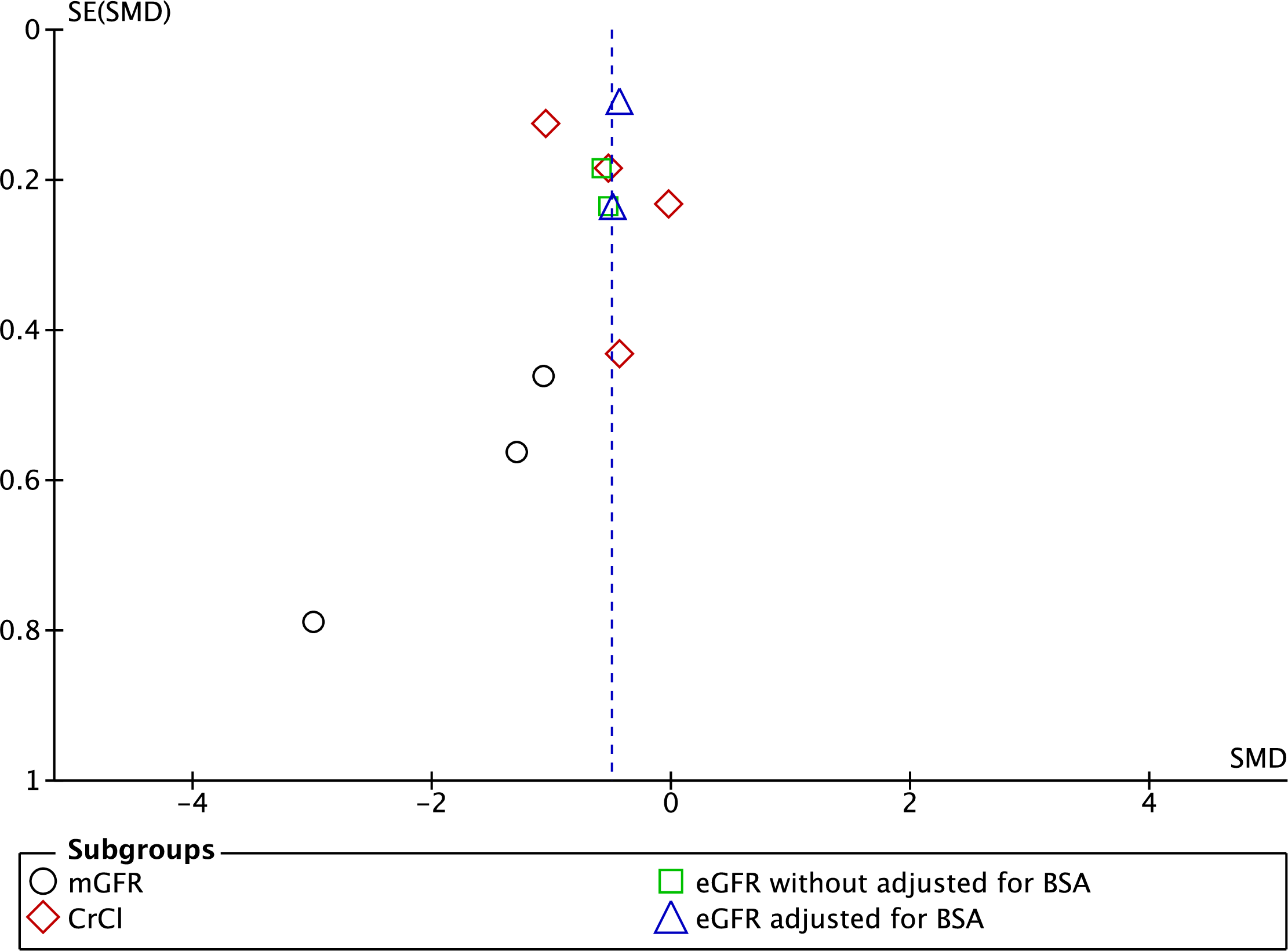

Supplement: S1 Fig — Funnel plot to assess publication for the most frequently reported outcome glomerular hyperfiltration. mGFR: measured glomerular filtration rate; eGFR: estimated glomerular filtration rate; Crcl: creatinine clearance; BSA: body surface area. (TIF) [file pone.0163907.s001.tif]

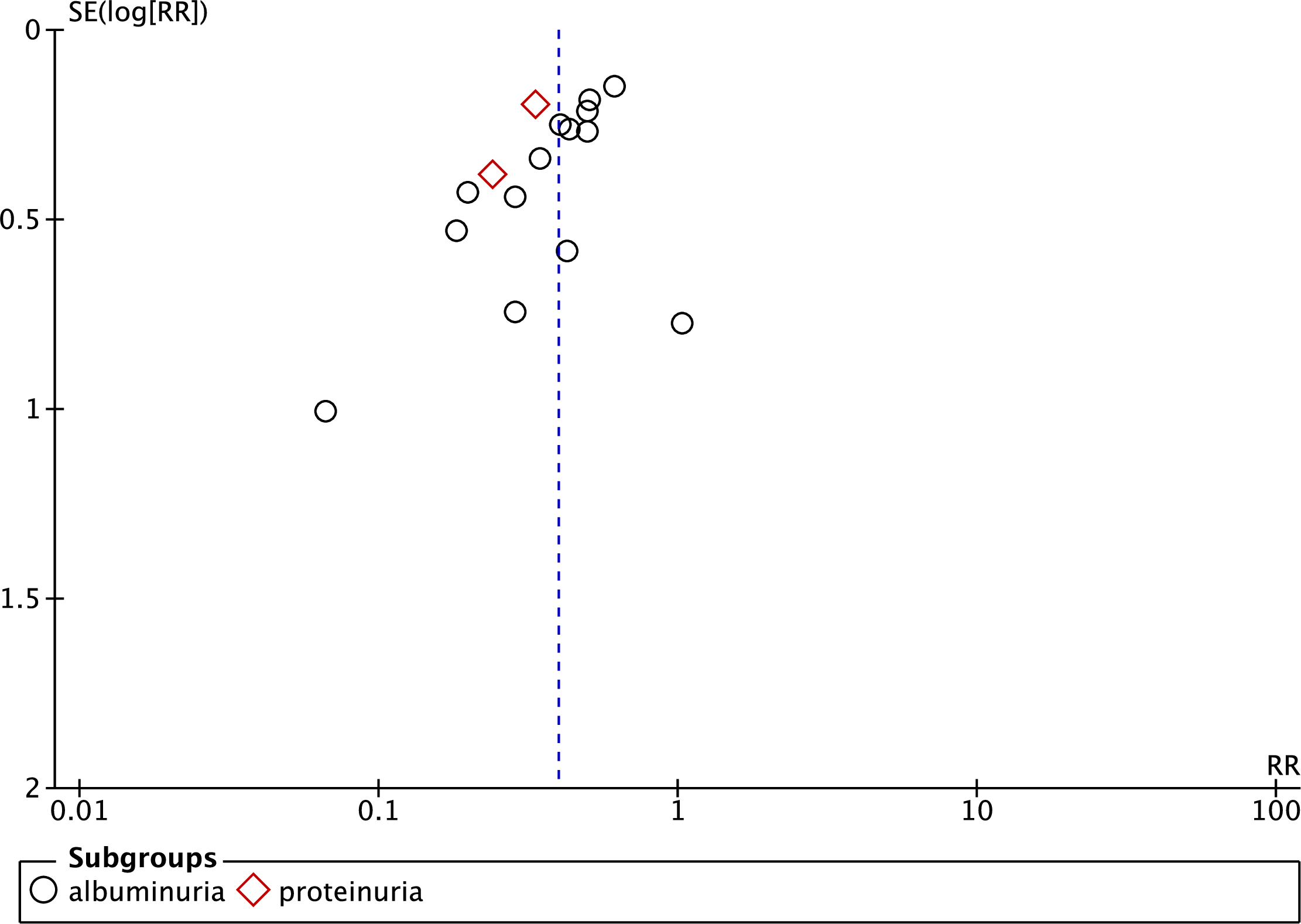

Supplement: S2 Fig — Funnel plot to assess publication for the most frequently reported outcome albuminuria and proteinuria. (TIF) [file pone.0163907.s002.tif]
